# The importance of multi-modal imaging and clinical information for humans and AI-based algorithms to classify breast masses (INSPiRED 003): an international, multicenter analysis

**DOI:** 10.1007/s00330-021-08519-z

**Published:** 2022-02-17

**Authors:** André Pfob, Chris Sidey-Gibbons, Richard G. Barr, Volker Duda, Zaher Alwafai, Corinne Balleyguier, Dirk-André Clevert, Sarah Fastner, Christina Gomez, Manuela Goncalo, Ines Gruber, Markus Hahn, André Hennigs, Panagiotis Kapetas, Sheng-Chieh Lu, Juliane Nees, Ralf Ohlinger, Fabian Riedel, Matthieu Rutten, Benedikt Schaefgen, Maximilian Schuessler, Anne Stieber, Riku Togawa, Mitsuhiro Tozaki, Sebastian Wojcinski, Cai Xu, Geraldine Rauch, Joerg Heil, Michael Golatta

**Affiliations:** 1 University Breast Unit, Department of Obstetrics and Gynecology, Heidelberg University Hospital, Im Neuenheimer Feld 440, 69120 Heidelberg, Germany; 2 MD Anderson Center for INSPiRED Cancer Care (Integrated Systems for Patient-Reported Data), The University of Texas MD Anderson Cancer Center, Houston, TX USA; 3 Department of Symptom Research, The University of Texas MD Anderson Cancer Center, Houston, TX USA; 4 Department of Radiology, Northeast Ohio Medical University, Ravenna, OH USA; 5 Department of Gynecology and Obstetrics, University of Marburg, Marburg, Germany; 6 Department of Gynecology and Obstetrics, University of Greifswald, Greifswald, Germany; 7 Department of Radiology, Institut Gustave Roussy, Villejuif Cedex, France; 8 Department of Radiology, University Hospital Munich-Grosshadern, Munich, Germany; 9 Department of Radiology, University of Coimbra, Coimbra, Portugal; 10 Department of Gynecology and Obstetrics, University of Tuebingen, Tuebingen, Germany; 11 Department of Biomedical Imaging and Image-Guided Therapy, Medical University of Vienna, Vienna, Austria; 12 Department of Radiology, Jeroen Bosch Hospital, ‘s-Hertogenbosch, The Netherlands; 13 Radboud University Medical Center, Nijmegen, The Netherlands; 14 National Center for Tumor Diseases, Heidelberg University Hospital, Heidelberg, Germany; 15 Department of Radiology, Sagara Hospital, Kagoshima, Japan; 16 Department of Gynecology and Obstetrics, Breast Cancer Center, Klinikum Bielefeld Mitte GmbH, Bielefeld, Germany; 17 Institute of Biometry and Clinical Epidemiology, Charité – Universitätsmedizin Berlin, Freie Universität Berlin, Humboldt-Universität Zu Berlin, Berlin , Germany

**Keywords:** Breast cancer, Ultrasonography, Machine learning, Artificial intelligence

## Abstract

**Objectives:**

AI-based algorithms for medical image analysis showed comparable performance to human image readers. However, in practice, diagnoses are made using multiple imaging modalities alongside other data sources. We determined the importance of this multi-modal information and compared the diagnostic performance of routine breast cancer diagnosis to breast ultrasound interpretations by humans or AI-based algorithms.

**Methods:**

Patients were recruited as part of a multicenter trial (NCT02638935). The trial enrolled 1288 women undergoing routine breast cancer diagnosis (multi-modal imaging, demographic, and clinical information). Three physicians specialized in ultrasound diagnosis performed a second read of all ultrasound images. We used data from 11 of 12 study sites to develop two machine learning (ML) algorithms using unimodal information (ultrasound features generated by the ultrasound experts) to classify breast masses which were validated on the remaining study site. The same ML algorithms were subsequently developed and validated on multi-modal information (clinical and demographic information plus ultrasound features). We assessed performance using area under the curve (AUC).

**Results:**

Of 1288 breast masses, 368 (28.6%) were histopathologically malignant. In the external validation set (*n* = 373), the performance of the two unimodal ultrasound ML algorithms (AUC 0.83 and 0.82) was commensurate with performance of the human ultrasound experts (AUC 0.82 to 0.84; *p* for all comparisons > 0.05). The multi-modal ultrasound ML algorithms performed significantly better (AUC 0.90 and 0.89) but were statistically inferior to routine breast cancer diagnosis (AUC 0.95, *p* for all comparisons ≤ 0.05).

**Conclusions:**

The performance of humans and AI-based algorithms improves with multi-modal information.

**Key Points:**

*• The performance of humans and AI-based algorithms improves with multi-modal information.*

*• Multimodal AI-based algorithms do not necessarily outperform expert humans.*

*• Unimodal AI-based algorithms do not represent optimal performance to classify breast masses.*

**Supplementary Information:**

The online version contains supplementary material available at 10.1007/s00330-021-08519-z.

## Introduction

The use of automated medical image analysis by AI-based algorithms has generated great enthusiasm: world-class radiological evaluations may become frequently available for low-income countries, rural areas, or physicians in training [1]. Moreover, the automated evaluation of images may help radiologists in managing the increasing workload demands [2]. Algorithms for medical image analysis are developed either by using hand-crafted image features (extracted automatically or by human readers) that are analyzed by machine learning algorithms or by using deep learning techniques that do not require prior feature extraction [1]. Such algorithms have already shown great diagnostic performance comparable to human expert readers in some areas [3]. However, a recent survey among the members of the American College of Radiology and the Radiological Society of North America showed that very few physicians use such imaging algorithms in their practice (about 30%, mainly for research purposes) and that among those, 93% reported inconsistent results of these algorithms in practice. About 95% said they would not put their faith into a diagnosis solely made by an algorithm (although some of them have FDA clearance) [4]. The discrepancy between the excellent performance reported by newly developed imaging algorithms and their non-use in clinical practice as well as the reluctance expressed by human imaging experts seems striking. An explanation for this may be that algorithms which are trained on image data alone may perform on par with human image readers when looking only at those images — but this does not represent the clinical reality in which imaging information (of multiple imaging modalities) is often considered alongside contextualizing clinical and demographic information.

Taking breast cancer diagnosis as an example, several imaging modalities (usually ultrasound and mammography, sometimes MRI) are used to evaluate indeterminate breast masses in combination with clinical and demographic information like patient age, suspicious palpability, disease history, and family medical history [5, 6]. Especially breast ultrasound has been under intense evaluation over the past years as it showed potential to identify cancers that are initially missed in mammography but ultrasound also leads to more false-positive findings [7]. The absent integration of contextualizing clinical and demographic information and of different imaging modalities into AI-based, diagnostic algorithms (especially in breast cancer diagnosis) may restrict the current performance of those diagnostic models. Although this knowledge gap has important implications for clinical practice, it has not been addressed systematically yet.

In this study, we compared the diagnostic performance of routine breast cancer diagnosis to breast ultrasound interpretations by humans or AI-based algorithms which were trained either on unimodal information (ultrasound features) or on multi-modal information (clinical and demographic information in addition to ultrasound features) to classify breast masses. We hypothesized that both humans and AI-based algorithms can improve their performance when considering multi-modal instead of unimodal information. For our analysis, we used data of an international multicenter trial that evaluated the use of a new ultrasound technique compared to traditional B-mode breast ultrasound [8].

## Material and methods

### Patient recruitment and selection

Patients were recruited as part of an international multicenter trial (NCT02638935). The trial was conducted at 12 study sites across 7 countries (Austria, France, Germany, Japan, Portugal, The Netherlands, the USA) from February 2016 to March 2019. Women aged 18 years or older who presented with an indeterminate breast mass ≥ 0.5 and ≤ 5 cm in largest diameter size in 2D B-mode ultrasound were enrolled. Only one mass per patient was included. As by requirement of the parental trial, all patients underwent histopathological confirmation.

### Design and definitions

In the clinical routine, a breast mass was classified as (potentially) benign or malignant after evaluating different imaging modalities (mammography, 2D B-mode ultrasound, and/or MRI, as applicable in clinical routine) alongside additional demographic and clinical information about the patients’ age, disease history, and family medical history. Three physicians specialized in ultrasound diagnosis from separate study sites performed a second read of all ultrasound images, without access to any clinical information on patients. The three ultrasound experts, who had 10 to 30 years of experience in breast cancer diagnosis, consisted of one radiology professor, one professor specialized in breast diagnosis (head of breast diagnosis), and one senior physician specialized in breast diagnosis (head of breast diagnosis).

The risk of malignancy was evaluated according to the American College of Radiology (ACR) BI-RADS criteria and a BI-RADS score was assigned for all patients in the clinical routine and by the ultrasound experts. BI-RADS assigns risk categories to breast masses: BI-RADS III is assigned for patients with a risk of malignancy > 0% but ≤ 2%, BI-RADS IV for > 2% but ≤ 95%, and BI-RADS V for > 95% risk of malignancy. To further refine this broad risk assessment, a continuous likelihood score of malignancy was assigned for all patients in addition to the BI-RADS score. Of the single variables that were considered to evaluate the risk of malignancy in the clinical routine, the single BI-RADS descriptors of the ultrasound evaluation, patient age, and palpability of the lesion were specifically documented for this trial.

For comparison, we developed and validated two machine learning (ML) algorithms trained on unimodal information (ultrasound features generated by the ultrasound experts, see Table 1) to classify breast masses. The same ML algorithms were subsequently trained on multi-modal information (clinical and demographic information in addition to ultrasound features). The full list of variables is shown in Table 1.

**Table 1:** Distribution of baseline and outcomes variables in the whole cohort and in the development and validation datasets

	Whole cohort (*n* = 1288)	Development set (*n* = 915)	Validation set (*n* = 373)	*p* value**
Patient age —yr. (SD)	46.5 (16.0)	47.0 (16.0)	45.3 (16.0)	0.089^#^
- < 50 years —no. (%)	785 (60.9)	549 (60.0)	236 (63.3)	0.304*
- ≥ 50 years —no. (%)	503 (39.1)	366 (40.0)	137 (36.7)	0.304*
Clinically suspicious palpability (*n* = 1288)				< 0.001*
No —no. (%)	631 (49.0)	495 (54.1)	136 (36.5)	
Yes —no. (%)	657 (51.0)	420 (45.9)	237 (63.5)	
Breast mass dimensions on B-mode breast ultrasound (*n* = 1288)				
Mass size in longest axis —mm. (SD)	31.2 (14.6)	30.9 (14.7)	31.8 (14.5)	0.317^#^
Mass size in perpendicular plane —mm. (SD)	19.9 (10.4)	18.1 (9.2)	24.2 (11.9)	< 0.001^#^
Mass size in orthogonal plane —mm. (SD)	23.8 (12.5)	24.6 (12.9)	22.0 (11.2)	< 0.001^#^
Tissue composition (*n* = 1288)				
Homogeneous background texture; fat —no. (%)	259 (20.1)	199 (21.7)	60 (16.1)	0.026*
Heterogeneous background texture —no. (%)	563 (43.7)	395 (43.2)	168 (45.0)	0.581*
Homogeneous background texture; fibroglandular —no. (%)	466 (36.2)	321 (35.1)	145 (38.9)	0.222*
Shape of mass (*n* = 1288)				
Oval —no. (%)	906 (70.3)	654 (71.5)	252 (67.6)	0.184*
Round —no. (%)	68 (5.3)	43 (4.7)	25 (6.7)	0.187*
Irregular —no. (%)	314 (24.4)	218 (23.8)	96 (25.7)	0.513*
Orientation of mass (*n* = 1288)				0.948
Parallel —No. (%)	1046 (81.2)	744 (81.3)	302 (81.0)	
Not parallel —No. (%)	242 (18.8)	171 (18.7)	71 (19.0)	
Margin of mass (*n* = 1288)				0.011
Circumscribed —No. (%)	619 (48.1)	461 (49.6)	158 (42.4)	
Non-Circumscribed —No. (%)	669 (51.9)	454 (49.6)	215 (57.6)	
- Microlobulated margin —no. (%)	69 (5.4)	48 (5.2)	21 (5.6)	0.888*
- Indistinct margin —no. (%)	573 (44.5)	391 (42.7)	182 (48.8)	0.054*
- Angular margin —no. (%)	99 (7.7)	71 (7.8)	28 (7.5)	0.969*
- Spiculated margin —no. (%)	19 (1.5)	13 (1.4)	6 (1.6)	1.00*
Echo pattern (*n* = 1288)				
Anechoic —no. (%)	2 (0.2)	0 (0.0)	2 (0.5)	0.151*
Complex cystic and solid —no. (%)	21 (1.6)	16 (1.7)	5 (1.3)	0.778*
Hypoechoic —no. (%)	1065 (82.7)	775 (84.7)	290 (77.7)	0.004*
Isoechoic —no. (%)	101 (7.8)	62 (6.8)	39 (10.5)	0.035*
Heterogeneous —no. (%)	90 (7.0)	57 (6.2)	33 (8.8)	0.121*
Hyperechoic —no. (%)	9 (0.7)	5 (0.5)	4 (1.1)	0.510*
Posterior features (*n* = 1288)				
None —no. (%)	759 (58.9)	522 (57.0)	237 (63.5)	0.037*
Enhancement —no. (%)	370 (28.7)	275 (30.1)	95 (25.5)	0.114*
Combined pattern —no. (%)	15 (1.2)	9 (1.0)	6 (1.6)	0.508*
Shadowing —no. (%)	144 (11.2)	109 (11.9)	35 (9.4)	0.227*
Calcification (*n* = 1288)				0.552
No calcification —no. (%)	1231 (95.6)	877 (95.8)	354 (94.9)	
Calcification —no. (%)	57 (4.4)	38 (4.2)	19 (5.1)	
Histopathological results (*n* = 1288)				0.010
Benign —no. (%)	920 (71.4)	673 (73.6)	247 (66.2)	
- Fibroadenoma —no. (%)	528 (57.5)	381 (72.2)	147 (27.8)	0.504*
- Lipoma —no. (%)	4 (0.4)	3 (75.0)	1 (25.0)	1*
- Atypia —no. (%)	1 (0.1)	1 (100)	0 (0.0)	1*
- Condense cyst —no. (%)	65 (7.1)	55 (84.6)	10 (15.4)	0.043*
- Other —no. (%)	320 (34.9)	231 (72.2)	89 (27.8)	0.708*
Malignant —no. (%)	368 (28.6)	242 (26.4)	126 (33.8)	
- No special type —no. (%)	250 (67.9)	154 (61.6)	96 (38.4)	0.020*
- Invasive lobular carcinoma —no. (%)	25 (6.8)	13 (52.0)	12 (48.0)	0.199*
- Invasive tubular carcinoma —no. (%)	7 (1.9)	7 (100)	0 (0.0)	0.127*
- Medullary carcinoma —no. (%)	5 (1.4)	2 (40.0)	3 (60.0)	0.455*
- Papillary carcinoma —no. (%)	12 (3.3)	11 (91.7)	1 (8.3)	0.107*
- Ductal carcinoma in situ —no. (%)	26 (7.1)	22 (84.6)	4 (15.4)	0.059*
- Other —no. (%)	43 (11.7)	33 (76.7)	10 (23.3)	0.149*

^*^ *p* values refer to chi-square tests for binary feature evaluation (feature true vs. feature not true)

^#^ *p* values refer to *t*-test to evaluate mean differences of continuous data

^**^ *p* values refer to differences in the development and validation sets

Following ACR BI-RADS guidelines, we assumed breast masses to be malignant when the risk of malignancy was equal to or above 2% according to BI-RADS 4 or 5. All patients underwent histopathologic confirmation against which the diagnostic predictions were compared.

### Algorithm development

Choice of algorithms and reporting on them were informed by guidelines on how to use ML in medicine [9], how to report findings of diagnostic tests [10], and multivariate prediction models [11] as well as previously published research by our group [12–15] We developed and validated two algorithms to predict malignancy of a breast mass:

1. Logistic regression (LR) with elastic net penalty: We chose this algorithm because of its ability to attenuate the influence of certain predictors on the model, leading to greater generalizability to new datasets [16, 17]. This algorithm is limited to identifying linear relations between the predictor variables and the outcome.
2. Extreme gradient boosting (XGBoost) tree: Gradient boosting refers to a machine learning technique in which the final prediction model consists of an ensemble of several, stepwise built models [18]. Gradient boosting is commonly applied to decision trees which results in an ensemble model combining the prediction of several trees. We chose this algorithm because of its ability to identify more complex, non-linear patterns in data while still being interpretable.

Algorithms were trained and tuned on the development set using tenfold cross-validation; a hypergrid-search was used for hyperparameter tuning (see Supplementary Appendix for optimal hyperparameters, the results of the internal testing, and data preparation steps). The final model was then externally validated using an independent dataset. As this was a large international multicenter trial, we selected one trial site as an independent validation dataset on which the final model was (externally) validated. Guidelines for multivariable risk prediction models recommend validation of such a model in a dataset of at least 100 events [11]. Only one trial site had at least 100 events and was thus used as validation set (study site 1 of the parental trial) [8]. The other 11 trial sites were used as a development set.

We provide a more detailed description of all algorithms and the algorithm development as well as a detailed evaluation of our study according to the abovementioned guidelines [9–11]. in the online Supplementary Appendix.

### Statistical analysis

Descriptive statistics including absolute and relative frequencies as well as chi-square tests for categorical data and mean and standard deviation were used alongside *t*-tests for continuous data to compare the distribution of baseline and outcome variables in the development and validation sets.

To assess the diagnostic performance in classifying breast masses of the clinical routine, the ultrasound experts, the unimodal and multi-modal ultrasound ML algorithms, area under the receiver-operating characteristics curve (AUC), and accompanying 95% confidence intervals were calculated for every model using 2000 bootstrap replicates that were drawn from the validation dataset and stratified for the outcome variable (malignant/benign). We conducted subgroups analyses to compare the AUC of the unimodal and multi-modal ultrasound ML models in the external validation set across different age groups (< 50 years, ≥ 50 years) and across different histopathologic subgroups (malignant vs. benign).

Additionally, we compared sensitivity, specificity, and negative- and positive-predictive values to the gold standard of histopathologic evaluation and against each other; we computed 95% Clopper-Pearson confidence intervals.

Calibration of the ML models was evaluated using calibration plots (observed vs. predicted probabilities [19]) and Spiegelhalter’s *Z* statistic [20].

No multiplicity adjustments against type-I-error inflation were performed. Thus, these analyses are of descriptive nature. All *p* values must be interpreted descriptively and have no confirmatory value. Analysis was conducted using R software, Version 3.6.1 (the “caret” package of R was used for the model development).

### Ethical considerations

The trial was approved by all respective ethical committees and all participants gave their written informed consent. The research reported in this article complies with the Declaration of Helsinki.

## Results

### Patient recruitment

A total of 1294 women were enrolled. Six were excluded from the analysis because no data on the pathologic evaluation was available. The remaining 1288 underwent full clinical breast evaluations including clinical examinations and multi-modal imaging (B-mode breast ultrasound, mammography, MRI as applicable in clinical routine) followed by histopathologic evaluation of the mass.

### Baseline demographic and clinical characteristics

Table 1 shows the distribution of baseline and outcome variables for the whole cohort and in the development and external validation datasets that were used for the algorithm development and validation. In the whole cohort, the mean age was 46 years (standard deviation 16.0) and 368 of 1288 breast masses (28.6%) were malignant as confirmed by histopathology. When comparing the development (*n* = 915) and the external validation (*n* = 373) datasets, the validation set had a significantly higher proportion of histopathologically malignant masses (33.8% vs. 26.4%, *p* = 0.010) and a higher proportion of masses with clinically suspicious palpability (63.5% vs. 45.9%, *p* < 0.001), as well as some variations in the tissue composition, mass margins, echo pattern, and posterior features in the B-mode ultrasound images.

### Diagnostic performance evaluation

Diagnostic performance metrics of the clinical routine, of the three ultrasound experts, of the unimodal ultrasound ML algorithms, and of the multi-modal ultrasound ML algorithms in the validation set are shown in Table 2. AUROC in the clinical routine was 0.95 (95% CI 0.93 to 0.97), for the multi-modal ultrasound LR with elastic net penalty algorithm 0.90 (95% CI 0.87 to 0.93), for the multi-modal ultrasound XGBoost tree algorithm 0.89 (95% CI 0.85 to 0.92), for the unimodal ultrasound LR with elastic net penalty algorithm 0.83 (95% CI 0.78 to 0.87), for the unimodal ultrasound XGBoost tree algorithm 0.82 (95% CI 0.77 to 0.86), and 0.82 (95% CI 0.77 to 0.87), 0.82 (95% CI 0.77 to 0.87), and 0.84 (95% CI 0.79 to 0.89) for the ultrasound experts 1–3, respectively.

**Table 2:** Diagnostic performance of routine clinical breast diagnosis, the three ultrasound experts, the unimodal ultrasound machine learning algorithms, and the multi-modal ultrasound machine learning algorithms in the validation set

	AUROC – value (95% CI)	Sensitivity – % (95% CI); no	Specificity – % (95% CI); no	Negative predictive value – % (95% CI); no	Positive predictive value – % (95% CI); no
Clinical routine	0.95 (0.93 – 0.97)	100 (97.1 – 100), 126 of 126	35.6 (29.7 – 41.9), 88 of 247	100 (95.9 – 100), 88 of 88	44.2 (38.6 – 50.2), 126 of 285
US expert 1	0.82 (0.77 – 0.87)	88.1 (81.1 – 93.2) 111 of 126	49.4 (43.0 – 55.8), 122 of 247	89.1 (82.6 – 93.7), 122 of 137	47.0 (40.5 – 53.6), 111 of 236
US expert 2	0.82 (0.77 – 0.87)	96.0 (91.0 – 98.7), 121 of 126	24.3 (19.1 – 30.1), 60 of 247	92.3 (83.0 – 97.5), 60 of 65	39.3 (33.8 – 45.0), 121 of 308
US expert 3	0.84 (0.79 – 0.89)	91.3 (84.9 – 95.6), 115 of 126	31.2 (25.4 – 37.4), 77 of 247	87.5 (78.7 – 93.6), 77 of 88	40.4 (34.6 – 46.3), 115 of 285
Unimodal ultrasound ML algorithms*					
Logistic regression with elastic net penalty	0.83 (0.78 – 0.87)	100 (97.1 – 100), 126 of 126	9.3 (6.0 – 13.6), 23 of 247	100 (85.2 – 100), 23 of 23	36.0 (31.0 – 41.3), 126 of 350
XGBoost tree	0.82 (0.77 – 0.86)	100 (97.1 – 100), 126 of 126	18.2 (13.6 – 23.6), 45 of 247	100 (92.1 – 100), 45 of 45	38.4 (33.1 – 43.9), 126 of 328
Multi-modal ultrasound ML algorithms**					
Logistic regression with elastic net penalty	0.90 (0.87 – 0.93)	100 (97.1 – 100), 126 of 126	27.1 (21.7 – 33.1), 67 of 247	100 (94.6—100), 67 of 67	41.2 (35.6 – 46.9), (126 of 306)
XGBoost tree	0.89 (0.85 – 0.92)	100 (97.1 – 100), 126 of 126	19.0 (14.3 – 24.5), 47 of 247	100 (92.5 – 100), 47 of 47	38.7 (33.3 – 44.2), 126 of 326

^*^ Trained on ultrasound features

^**^ Trained on ultrasound features as well as patient age and palpability

*AUROC*, area under the receiver operating characteristic curve; *CI*, confidence interval; *ML*, machine learning; *US*, ultrasound

**Fig. 1:**
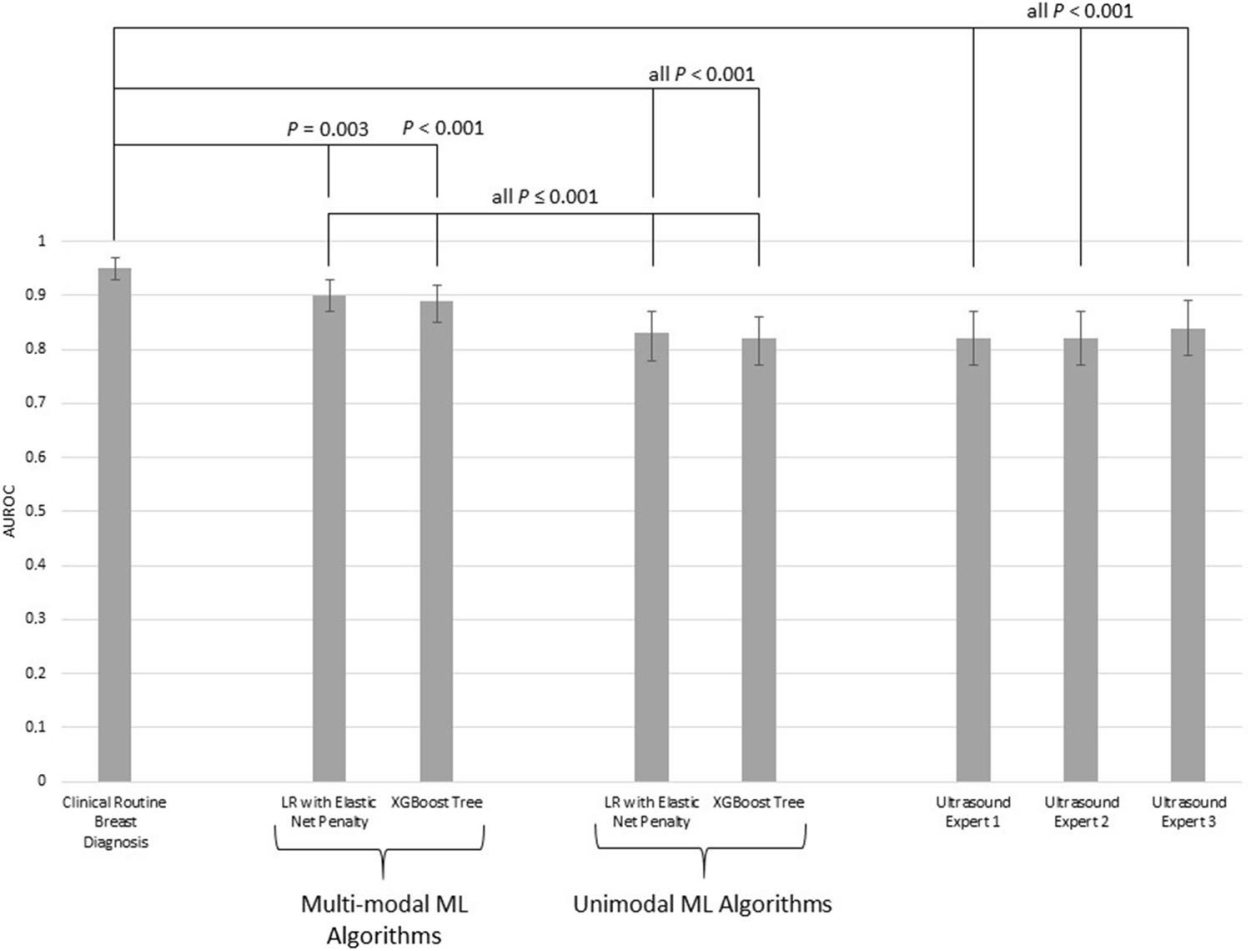
Performance comparison between the clinical routine, the ultrasound experts, the unimodal machine learning algorithms, and the multi-modal machine learning algorithms

**Fig. 2:**
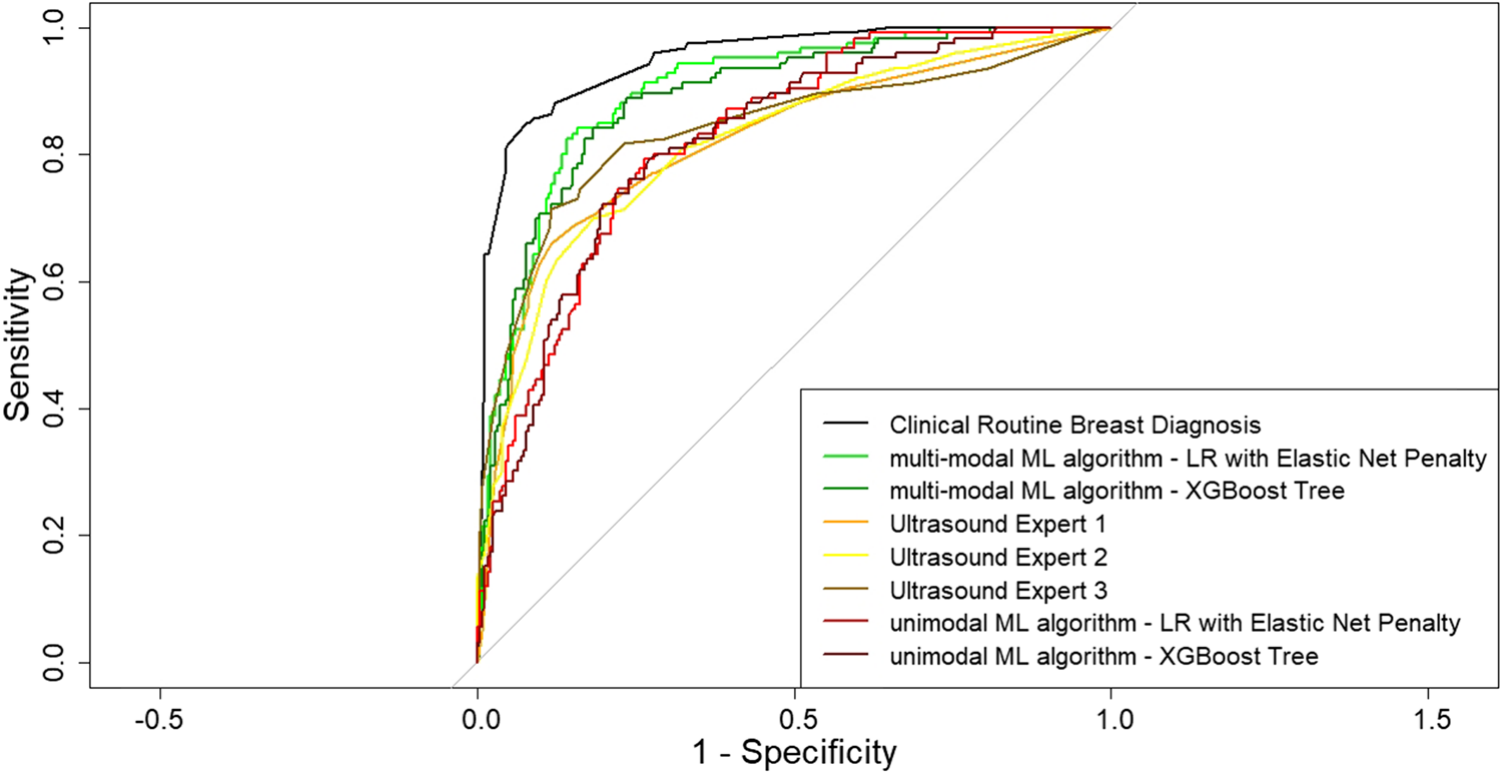
Receiver operating characteristic curves of the clinical routine, the ultrasound experts, the unimodal machine learning algorithms, and the multi-modal machine learning algorithms

Figure 1 summarizes the comparison in diagnostic performance between the different approaches: the performance of the unimodal ultrasound ML algorithms did not differ significantly from the performance of the ultrasound experts (*p* 0.361 to 0.935); the multi-modal ultrasound ML algorithms performed significantly better compared to all ultrasound experts and the unimodal algorithms (all *p* < 0.001); the clinical routine breast diagnosis performed significantly better compared to all other approaches (all *p* < 0.01). Corresponding ROC curves are illustrated in Fig. 2.

Calibration plots of the ML models are illustrated in Supplemental Fig. 1 and indicate well-calibrated models, which was confirmed by Spiegelhalter’s Z (good calibration in 3 out of 4 models, see Supplementary Appendix). The unimodal LR with elastic net penalty showed an impaired calibration for mid-range probabilities of malignancy.

### Insights into model predictions and traditional multivariable logistic regression

Predictive coefficients of the unimodal and multi-modal LR with elastic net penalty are listed in Table 3. For the multi-modal algorithm, patient age was the most important predictor of malignancy (regularized *ß* = 7.60, 95% CI 7.53 to 7.73), followed by spiculated margins (regularized *ß* = 1.10, 95% CI 0.21 to 1.99), a not parallel orientation of the mass (regularized *ß* = 0.88, 95% CI 0.52 to 1.24), clinically suspicious palpability (regularized *ß* = 0.84, 95% CI 0.56 to 1.28), and an irregular shape (regularized *ß* = 0.54, 95% CI 0.0 to 1.08).

**Table 3:** Predictive coefficients of the uni- and multi-modal logistic regression with elastic net penalty algorithms

	Regularized coefficients* for unimodal algorithm (95% CI)	Regularized coefficients* for multi-modal algorithm (95% CI)
Clinical information		
Patient age	NA	7.60 (7.53 to 7.73)
Clinically suspicious palpability	NA	0.84 (0.56 to 1.28)
Breast mass dimensions on B-mode breast ultrasound		
Mass size in longest axis	− 0.51 (− 0.58 to − 0.43)	− 1.04 (− 1.11 to − 0.96)
Mass size in perpendicular plane	0.65 (0.54 to 0.75)	1.50 (1.41 to 1.60)
Mass size in orthogonal plane	− 0.15 (− 0.09 to − 0.21)	0.00 (− 0.06 to 0.06)
Tissue composition		
Homogeneous background texture; fat	0.09 (− 0.42 to 0.61)	− 0.01 (− 0.53 to 0.51)
Heterogeneous background texture	0.0 (− 0.45 to 0.46)	0.00 (− 0.45 to 0.46)
Homogeneous background texture; fibroglandular	− 0.07 (− 0.76 to 0.62)	0.00 (− 0.69 to 0.70)
Shape of mass		
Oval	− 0.35 (− 0.65 to − 0.06)	− 0.04 (− 0.34 to 0.31)
Round	− 0.01 (− 0.48 to 0.40)	− 0.32 (− 0.73 to − 0.09)
Irregular	0.42 (− 0.12 to 0.96)	0.54 (0.0 to 1.08)
Orientation of mass		
Not parallel	0.61 (0.25 to 0.97)	0.88 (0.52 to 1.24)
Margin of mass		
Non-circumscribed	0.45 (0.11 to 0.79)	0.55 (0.21 to 0.89)
Microlobulated margin	0.50 (0.29 to 0.71)	0.62 (0.41 to 0.83)
Indistinct margin	0.43 (− 0.41 to 1.24)	0.51 (− 0.33 to 1.35)
Angular margin	0.50 (0.24 to 0.76)	0.55 (0.29 to 0.81)
Spiculated margin	0.96 (0.07 to 1.85)	1.10 (0.21 to 1.99)
Echo pattern		
Anechoic	0.0 (− 2.3 to 2.3)	0.0 (− 2.3 to 2.3)
Complex cystic and solid	0.07 (− 0.52 to 0.69)	0.11 (− 0.51 to 0.73)
Hypoechoic	0.0 (− 0.7 to 0.7)	0.0 (− 0.6 to 0.7)
Isoechoic	0.13 (− 0.33 to 0.59)	0.20 (− 0.26 to 0.66)
Heterogeneous	− 0.05 (− 0.56 to 0.46)	− 0.23 (− 0.74 to 0.28)
Hyperechoic	− 0.32 (− 1.03 to 0.41)	− 0.58 (− 1.61 to 0.15)
Posterior features		
None	− 0.09 (− 0.33 to 0.15)	− 0.20 (− 0.44 to 0.04)
Enhancement	0.0 (− 0.81 to 0.81)	0.0 (− 0.80 to 0.81)
Combined pattern	− 0.50 (− 1.62 to 0.62)	− 0.84 (− 1.96 to 0.18)
Shadowing	0.31 (0.14 to 0.48)	0.26 (0.09 to 0.43)
Calcification		
Calcification	0.48 (0.25 to 0.61)	0.76 (0.53 to 0.99)

^*^ Positive values indicate a positive association with malignancy

**Fig. 3:**
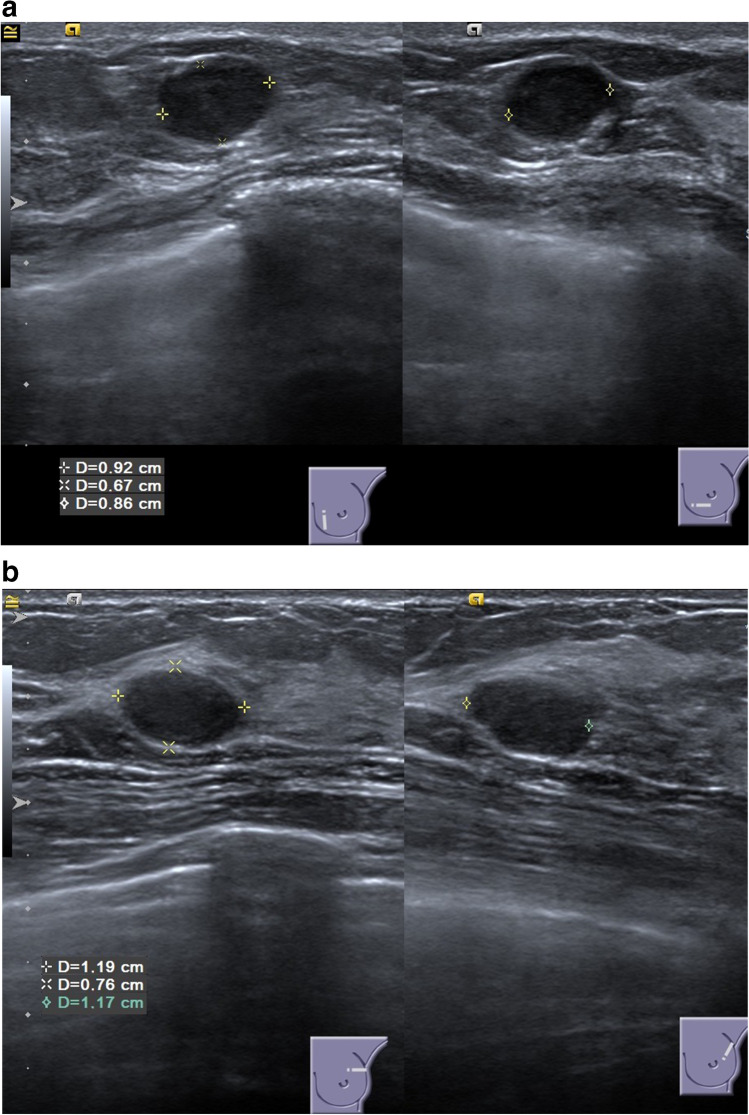
Ultrasound Images. **a** This patient’s ultrasound images were evaluated to show a benign breast mass by the three ultrasound experts but to show a malignant breast mass by full clinical breast evaluation. This patient was 41 years old with a positive family history for breast cancer and a clinically suspicious palpable tumor. Histopathology showed a luminal B, NST, G3 carcinoma. **b** This patient’s ultrasound images were evaluated to show a benign breast mass by the three physician experts and by full clinical breast evaluation. This patient was 25 years old without any clinically suspicious signs. Histopathology showed a fibroadenoma

Figure 3 illustrates insights into the variable importance for the predictions made by the XGBoost tree via Shapley Additive Explanations (SHAP) values.

For comparison, odds ratios of a traditional multivariable logistic regression are listed in Table 4.

**Table 4:** Traditional multivariable logistic regression

	Odds ratio (95% CI)	*p* value
Clinical information		
Patient age	1.10 (1.09 – 1.12)	< 0.001
Clinically suspicious palpability		
No	1 [reference]	
Yes	3.90 (2.59 – 5.94)	< 0.001
Breast mass dimensions on B-mode breast ultrasound		
Mass size in longest axis	0.89 (0.82 – 0.95)	0.001
Mass size in perpendicular plane	1.12 (1.05 – 1.20)	< 0.001
Mass size in orthogonal plane	1.05 (1.00 – 1.12)	0.098
Tissue composition		
Homogeneous background texture; fat	1 [reference]	
Heterogeneous background texture	1.21 (0.76 – 1.93)	0.432
Homogeneous background texture; fibroglandular	1.02 (0.61 – 1.69)	0.947
Shape of mass		
Oval	1 [reference]	
Round	0.86 (0.39 – 1.84)	0.697
Irregular	1.36 (0.82 – 2.27)	0.232
Orientation of mass		
Parallel	1 [reference]	
Not parallel	2.66 (1.59 – 4.48)	< 0.001
Margin of mass		
Circumscribed	1 [reference]	
Non-circumscribed	2.67 (1.27 – 5.55)	0.009
Microlobulated margin	2.42 (1.17 – 5.04)	0.017
Indistinct margin	1.42 (0.74 – 2.80)	0.298
Angular margin	2.11 (1.08 – 4.21)	0.031
Spiculated margin	2.69 (0.44 – 5.25)	0.372
Echo pattern		
Anechoic	1 [reference]	
Complex cystic and solid	-	-
Hypoechoic	-	-
Isoechoic	-	-
Heterogeneous	-	-
Hyperechoic	-	-
Posterior features		
None	1 [reference]	
Enhancement	1.40 (0.88 – 2.21)	0.152
Combined pattern	1.03 (0.16 – 5.07)	0.969
Shadowing	2.17 (1.23 – 3.83)	0.007
Calcification		
No	1 [reference]	
Yes	3.08 (1.42 – 6.70)	< 0.001

“-” variable did not converge

### Subgroup analyses

We evaluated the diagnostic performance of the multi-modal ultrasound ML models in the external validation set across different patient subgroups (see Supplemental Table 1). The algorithms performed equally well across different age groups (< 50 years and ≥ 50 years, *p* > 0.05). The algorithms showed higher performance among patients with benign compared to malignant histopathology (*p* < 0.05). Detailed AUC values are listed in Supplemental Table 1.

### Further analyses

Table 5 shows the diagnostic performance of the clinical routine and of the three ultrasound experts in the whole cohort of 1288 patients. Their performance in the whole cohort and in the validation set did not differ significantly.

**Table 5:** Diagnostic performance of the clinical routine and of the three ultrasound experts in the whole cohort (*n* = 1288)

	Clinical routine*	Ultrasound expert 1	Ultrasound expert 2	Ultrasound expert 3
AUC, whole cohort (95% CI)	0.94 (0.92–0.95)	0.76 (0.73–0.79)	0.79 (0.76–0.82)	0.82 (0.79–0.85)
AUC, validation set (95% CI)	0.95 (0.93 to 0.97)	0.82 (0.77 to 0.87)	0.82 (0.77 to 0.87)	0.84 (0.79 to 0.89)
Performance difference compared to validation set —*p* value	0.390	0.121	0.659	0.739
Sensitivity —% (no.)	98.4% (362 of 368)	85.6% (315 of 368)	94.8% (349 of 368)	78.8% (290 of 368)
Specificity —% (no.)	46.2% (425 of 920)	41.4% (381 of 920)	21.5% (198 of 920)	44.9% (413 of 920)
Negative predictive value —% (no.)	98.6% (425 of 431)	87.8% (381 of 434)	91.2% (198 of 217)	84.1% (413 of 491)
Positive predictive value —% (no.)	42.2% (362 of 857)	36.9% (315 of 854)	32.6% (349 of 1071)	36.4% (290 of 797)

^*^ Evaluation of different imaging modalities (mammography, 2D B-mode ultrasound, and/or MRI, as applicable in clinical routine) alongside additional demographic and clinical information about the patients’ age, disease history, and family medical history

### Exemplary images

**Fig. 4:**
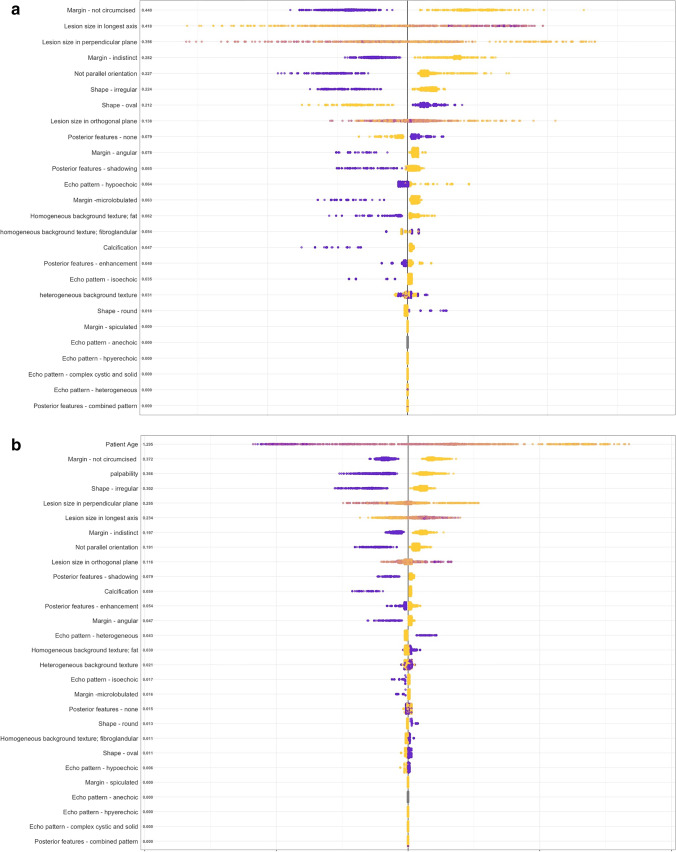
Shapley Additive Explanations (SHAP) Value Summary Plot of the Extreme Gradient Boosting (XGBoost) Tree Model. **a** XGboost – unimodal Algorithm. SHAP values on the left side of the x-axis indicate that the variable was important for predicting malignancy; values on the right side indicate that the variable was important for predicting a benign breast mass. Purple indicates a high variable value (e.g. margin – non-circumscribed: yes); yellow indicates a low variable value (e.g age: lower patient age). The values on the y-axis represent the overall global variable importance. **b** XGboost – multi-modal Algorithm

Ultrasound images of two exemplary patients are shown in Fig. 4 and illustrate the importance of contextualizing demographic and clinical patient information.

## Discussion

In this study, we compared the diagnostic performance of routine breast cancer diagnosis to breast ultrasound interpretations by humans or AI-based algorithms which were trained either on unimodal information (ultrasound features) or on multi-modal information (clinical and demographic information in addition to ultrasound features) to classify breast masses. Our classification algorithms showed equivalent or better performance compared to human readers in the classification of breast masses on ultrasound images. We show that beyond-human performance on imaging classification tasks does not necessarily yield state-of-the-art diagnostic decisions when compared to physicians who can evaluate multiple imaging sources alongside other relevant demographic and clinical information. We demonstrate that AI-based algorithms, like humans, can improve diagnostic accuracy of breast cancer classification by considering image data in combination with data on individuals’ demographics and clinical status. Contextualizing clinical and demographic information is a key element in the diagnostic pathway — even when imaging interpretation is optimized or enhanced by AI-based algorithms, there is an inherent limitation in accuracy of using only one imaging modality for breast cancer diagnosis. Further work is warranted to develop and evaluate individualized diagnostic models which combine imaging with comprehensive clinical and demographic data to better represent the diagnostic pathway of routine clinical breast diagnosis.

In interpreting our findings, some points should be further discussed. First, even the most AI-based imaging algorithms might be limited when evaluating only images of one imaging modality without contextualizing clinical and demographic information. This becomes evident when looking at the two exemplary patients whose ultrasound images are illustrated in Fig. 4. Moreover, a recent systematic review on AI-based image analysis identified 9 studies in the field of breast imaging [3]. Of these 9 studies, all reported that the developed algorithm showed a diagnostic performance comparable to that of human experts but all compared the performance against human image readers and not against full diagnostic evaluations in the clinical routine, all algorithms were trained on unimodal imaging information (7 ultrasound, 2 mammography), and only 3 were externally validated [21–29]. Analyzing contextualizing patient information for complex risk assessments by AI-based algorithms has yielded promising results in other fields [13–15] Thus, the absent integration of contextualizing clinical and demographic information and of different imaging modalities into AI-based, diagnostic algorithms (especially in breast cancer diagnosis) may not only restrict the current performance of those models —the common claim that some of these models have already achieved a diagnostic performance similar to human experts could also give clinicians a false sense of security when using image algorithms that have, however, not yet been (prospectively) compared against clinical routine performance. Moreover, AI-based algorithms in the field of breast imaging are often compared to the categorical BI-RADS assessment. As AI-based algorithms produce a continuous risk of malignancy as output, this may inherently lead to a higher performance when comparing AI-based algorithms with BI-RADS categories. In our study, a continuous likelihood score of malignancy was assigned for all patients in addition to the BI-RADS score. While this approach is not validated and may still lead to bias towards higher performance for AI-based algorithms it may enable a fairer comparison between AI-based algorithms and the BI-RADS categories assigned by humans.

Second, our multi-modal ultrasound ML algorithms were trained on image features as well as clinical and demographic information, but the amount of documented, explainable information was limited to ultrasound features, patient age, and clinically suspicious palpability. Further work is warranted to develop (more reliable) diagnostic models which combine imaging with comprehensive clinical and demographic data to fully represent the clinical reality (see commonly considered variables according to the National Comprehensive Cancer Network guideline for breast cancer screening [5]). Moreover, current research evaluates the feasibility of automated breast ultrasound and its combination with digital breast tomosynthesis which may further advance (automated) multi-modal breast image analysis in the future [30, 31].

Third, relying on traditional group-level associations may contribute to the ongoing discussion about high false-positive rates in breast diagnosis [32], which was also observed in our study (46% specificity for the clinical routine assessment in the whole cohort, Table 5). Individualized predictions by complex risk models may help improve diagnostic accuracy to avoid physical and psychological distress for patients and reduce treatment burden for providers and healthcare systems.

Fourth, algorithms for medical image analysis or classification are developed either by using hand-crafted image features that are analyzed by ML algorithms or by using deep learning techniques that do not require prior feature extraction [1]. In our study, we used the first approach. Although deep learning techniques showed great potential for automated medical image analysis in the past decade, they commonly do not outperform humans in image detection or classification tasks [3]. In fact, for some classification tasks, analyzing hand-crafted image features showed to be superior to deep learning approaches in small- to medium-sized datasets [33]. As the aim of our present analysis was to demonstrate the inherent limitations of developing AI-based algorithms on unimodal instead of multi-modal information and comparing their performance against image readers instead of clinical routine decisions, we do not expect the choice of feature-based machine learning instead of deep learning algorithms to limit our findings.

Fifth, our ultrasound experts performed a second read of all ultrasound images instead of performing the examination themselves. Although some evidence suggests that the interpretation of dynamic videos versus static images does not impair diagnostic performance, this may have caused some bias in our study and may have influenced the performance of the ultrasound experts [34].

## Conclusions

We show that beyond-human performance on imaging classification tasks does not necessarily yield state-of-the-art diagnostic decisions when compared to physicians who can evaluate multiple imaging sources alongside other relevant demographic and clinical information. AI-based algorithms that are not developed on multi-modal routine information (imaging, demographic, and clinical information) and that are subsequently not compared to the performance of this clinical routine may not represent state-of-the-art diagnostic performance. Confidence in AI-based algorithms that rely on solely one imaging modality may result in a misleading sense of security among clinicians. Further work is warranted to develop and evaluate individualized diagnostic models which combine imaging with comprehensive clinical and demographic data to better represent the diagnostic pathway of routine clinical breast diagnosis.

## Supplementary Information

Below is the link to the electronic supplementary material.

Supplementary file1 (DOCX 83 KB)

## Abbreviations

ACR: American College of Radiology
AUC: Area under the curve
BI-RADS: Breast Imaging Reporting and Data System
CI: Confidence interval
LR: Logistic regression
ML: Machine learning
ROC: Receiver-operating characteristic
XGBoost Tree: Extreme Gradient Boosting Tree

## Declarations

### Guarantor

The scientific guarantor of this publication is Michael Golatta.

### Statistics and biometry

Several of the authors have significant statistical expertise (Geraldine Rauch and Chris Sidey-Gibbons).

### Study subjects or cohorts overlap

Some study subjects or cohorts have been previously reported in:

Golatta et al: The Potential of Shear Wave Elastography to reduce unnecessary Biopsies in Breast Cancer Diagnosis: An international, diagnostic, multicenter Trial.” Accepted June 28th, 2021.

### Methodology

- • prospective
- • diagnostic or prognostic study
- • multicenter study

## References

[1] Hosny A, Parmar C, Quackenbush J, Schwartz LH, Aerts HJWL (2018). Artificial intelligence in radiology. Nat Rev Cancer.

[2] McDonald RJ, Schwartz KM, Eckel LJ (2015). The effects of changes in utilization and technological advancements ofcross-sectional imaging onradiologist workload. Acad Radiol.

[3] Liu X, Faes L, Kale AU (2019). A comparison of deep learning performance against health-care professionals in detecting diseases from medical imaging: a systematic review and meta-analysis. Lancet Digit Heal.

[4] American College of Radiology. Subject: (Docket No. FDA-2019-N-5592) “Public Workshop - Evolving Role of Artificial Intelligence in Radiological Imaging;” Comments of the American College of Radiology. https://www.acr.org/-/media/ACR/NOINDEX/Advocacy/acr_rsna_comments_fda-ai-evolvingrole-ws_6-30-2020.pdf. Published 2020. Accessed 3 Apr 2021

[5] National Comprehensive Cancer Network (2020). Breast cancer screening and diagnosis.

[6] Wöckel A, Festl J, Stüber T et al (2018) Interdisciplinary screening, diagnosis, therapy and follow-up of breast cancer. Guideline of the DGGG and the DKG (S3-Level, AWMF Registry Number 032/045OL, December 2017) - Part 1 with Recommendations for the Screening, Diagnosis and Therapy of Breast Cancer. Geburtshilfe Frauenheilkd. 78(10):927–948. 10.1055/a-0646-4522 10.1055/a-0646-4522 PMC6202580 30369626

[7] Yang L, Wang S, Zhang L (2020). Performance of ultrasonography screening for breast cancer: a systematic review and meta-analysis. BMC Cancer.

[8] Golatta M, Pfob A, Büsch C (2021). The potential of shear wave elastography to reduce unnecessary biopsies in breast cancer diagnosis: an international, diagnostic, multicenter trial. Ultraschall Med.

[9] Liu Y, Chen PHC, Krause J, Peng L (2020). How to read articles that use machine learning: users’ guides to the medical literature. JAMA.

[10] Cohen JF, Korevaar DA, Altman DG (2016). STARD 2015 guidelines for reporting diagnostic accuracy studies: explanation and elaboration. BMJ Open.

[11] Collins GS, Reitsma JB, Altman DG, Moons KGM (2015). Transparent reporting of a multivariable prediction model for individual prognosis or diagnosis (TRIPOD): the TRIPOD statement. Ann Intern Med.

[12] Sidey-Gibbons JAM, Sidey-Gibbons CJ (2019). Machine learning in medicine: a practical introduction. BMC Med Res Methodol.

[13] Pfob A, Sidey-Gibbons C, Lee H-B (2021). Identification of breast cancer patients with pathologic complete response in the breast after neoadjuvant systemic treatment by an intelligent vacuum-assisted biopsy. Eur J Cancer.

[14] Pfob A, Mehrara BJ, Nelson JA, Wilkins EG, Pusic AL, Sidey-Gibbons C (2021). Towards patient-centered decision-making in breast cancer surgery. Ann Surg.

[15] Sidey-Gibbons C, Pfob A, Asaad M (2021). Development of machine learning algorithms for the prediction of financial toxicity in localized breast cancer following surgical treatment. JCO Clin Cancer Inform.

[16] Tibshirani R (1997) The lasso method for variable selection in the Cox model. Stat Med 16(4):385–395. 10.1002/(sici)1097-0258(19970228)16:4<385::aid-sim380>3.0.co;2-3 10.1002/(sici)1097-0258(19970228)16:4<385::aid-sim380>3.0.co;2-3 9044528

[17] Friedman J, Hastie T, Tibshirani R (2010). Regularization paths for generalized linear models via coordinate descent. J Stat Softw.

[18] Friedman JH (2001). Greedy function approximation: a gradient boosting machine. Ann Stat.

[19] Harrell FE, Lee KL, Mark DB (1996) Multivariable prognostic models: Issues in developing models, evaluating assumptions and adequacy, and measuring and reducing errors. Stat Med 15(4):361–387. 10.1002/(SICI)1097-0258(19960229)15:4<361::AID-SIM168>3.0.CO;2-4 10.1002/(SICI)1097-0258(19960229)15:4<361::AID-SIM168>3.0.CO;2-4 8668867

[20] Spiegelhalter DJ (1986). Probabilistic prediction in patient management and clinical trials. Stat Med.

[21] Gargouri Ben Ayed N, Dammak Masmoudi A, Sellami D, Abid R (2015) New developments in the diagnostic procedures to reduce prospective biopsies breast. In: 2015 International Conference on Advances in Biomedical Engineering, ICABME 2015. Institute of Electrical and Electronics Engineers Inc.; 2015:205–208. 10.1109/ICABME.2015.7323288

[22] Becker AS, Mueller M, Stoffel E, Marcon M, Ghafoor S, Boss A. Classification of breast cancer in ultrasound imaging using a generic deep learning analysis software: a pilot study. Br J Radiol. 2018;91(1083) 10.1259/bjr.20170576 10.1259/bjr.20170576 PMC5965470 29215311

[23] Lin CM, Hou YL, Chen TY, Chen KH (2014). Breast nodules computer-aided diagnostic system design using fuzzy cerebellar model neural networks. IEEE Trans Fuzzy Syst.

[24] Kim SM, Han H, Park JM (2012). A comparison of logistic regression analysis and an artificial neural network using the BI-RADS lexicon for ultrasonography in conjunction with introbserver variability. J Digit Imaging.

[25] Fujioka T, Kubota K, Mori M (2019). Distinction between benign and malignant breast masses at breast ultrasound using deep learning method with convolutional neural network. Jpn J Radiol..

[26] Choi JS, Han BK, Ko ES (2019). Effect of a deep learning framework-based computer-aided diagnosis system on the diagnostic performance of radiologists in differentiating between malignant and benign masses on breast ultrasonography. Korean J Radiol.

[27] Byra M, Galperin M, Ojeda-Fournier H (2019). Breast mass classification in sonography with transfer learning using a deep convolutional neural network and color conversion. Med Phys.

[28] Becker AS, Marcon M, Ghafoor S, Wurnig MC, Frauenfelder T, Boss A (2017). Deep learning in mammography diagnostic accuracy of a multipurpose image analysis software in the detection of breast cancer. Invest Radiol.

[29] Stoffel E, Becker AS, Wurnig MC (2018). Distinction between phyllodes tumor and fibroadenoma in breast ultrasound using deep learning image analysis. Eur J Radiol Open.

[30] Golatta M, Franz D, Harcos A (2013). Interobserver reliability of automated breast volume scanner (ABVS) interpretation and agreement of ABVS findings with hand held breast ultrasound (HHUS), mammography and pathology results. Eur J Radiol.

[31] Schäfgen B, Juskic M, Radicke M (2020). Evaluation of the FUSION-X-US-II prototype to combine automated breast ultrasound and tomosynthesis. Eur Radiol.

[32] Le MT, Mothersill CE, Seymour CB, Mcneill FE (2016). Is the false-positive rate inmammography in North America too high?. Br J Radiol..

[33] Lin W, Hasenstab K, Moura Cunha G, Schwartzman A (2020). Comparison of handcrafted features and convolutional neural networks for liver MR image adequacy assessment. Sci Rep.

[34] Youk JH, Jung I, Yoon JH, et al. Comparison of inter-observer variability and diagnostic performance of the Fifth Edition of BI-RADS for breast ultrasound of static versus video images. Ultrasound Med Biol. 2016;42(9):2083–2088. 10.1016/j.ultrasmedbio.2016.05.006 10.1016/j.ultrasmedbio.2016.05.006 27324292

