# Supplementary material for: The importance of multi-modal imaging and clinical information for humans and AI-based algorithms to classify breast masses (INSPiRED 003): an international, multicenter analysis
Source: Eur Radiol. 2022 Feb 17;32(6):4101–15. doi: 10.1007/s00330-021-08519-z (PMC9123064; doi:10.1007/s00330-021-08519-z)

Supplemental Table 1. Diagnostic performance of the multi-modal machine learning models in the external validation set across different patient subgroups.

|  | Logistic Regression with elastic net Penalty, AUROC – value (95% CI) | *P* value | XGBoost Tree, AUROC – value (95% CI) | *P* value |
| --- | --- | --- | --- | --- |
| Age |  | 0.297 |  | 0.417 |
| <50 years | 0.92 (0.85-0.98) |  | 0.90 (0.93-0.96) |  |
| ≥50 years | 0.87 (0.78-0.95) |  | 0.84 (0.75-0.91) |  |
| Histopathology |  | 0.001* |  | <0.001 |
| benign | 0.97 (0.94-0.99) |  | 0.99 (0.94-1) |  |
| - fibroadenoma | 0.95 (0.90-0.98) |  | 0.98 (0.96-1) |  |
| - lipoma | -- | -- | -- | -- |
| - atypia | -- | -- | -- | -- |
| - condense cyst | 0.89 (0.67-1) |  | 0.89 (0.67-1) |  |
| - other | 0.81 (0.73-0.87) |  | 0.69 (0.60-0.78) |  |
| malignant | 0.86 (0.79-0.91) |  | 0.80 (0.73-0.86) |  |
| - no special type | 0.87 (0.80-0.94) |  | 0.80 (0.72-0.87) |  |
| - invasive lobular carcinoma | 0.87 (0.81-0.94) |  | 0.78 (0.69-0.86) |  |
| - invasive tubular carcinoma | -- | -- | -- | -- |
| - medullary carcinoma | -- | -- | -- | -- |
| - papillary carcinoma | -- | -- | -- | -- |
| - ductal carcinoma in-situ | -- | -- | -- | -- |
| - other | 0.87 (0.80-0.94) |  | 0.80 (0.71-0.87) |  |
|  |  |  |  |  |
|  |  |  |  |  |

* for benign versus malignant subgroup

-- = not calculated due to low number of patients

AUROC = Area under the receiver operating characteristic curve; CI = confidence interval

ML = Machine Learning

Supplemental Figure 1. Calibration Plots of the Machine Learning Models.

grey line: Logistic Regression with Elastic Net Penalty – unimodal Algorithm; black line: XGBoost Ultrasound Model – unimodal Algorithm; brown line: Logistic Regression with Elastic Net Penalty – multi-modal Algorithm; blue line: XGBoost – multi-modal Algorithm.


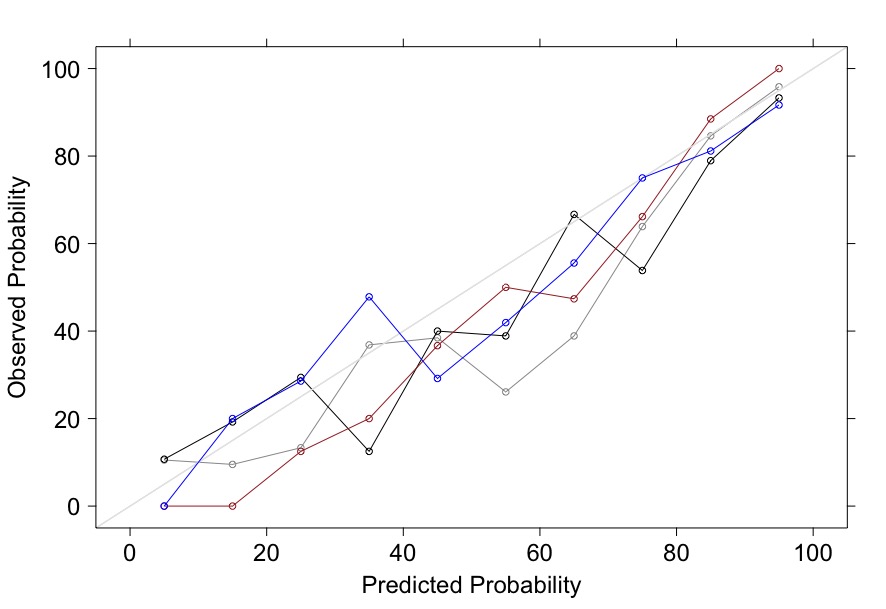

Supplement: Supplementary file 1 — Supplementary file1 (DOCX 83 KB) [file 330_2021_8519_MOESM1_ESM.docx]
